# Pseudoaneurysm in the anterior tibial artery after arthroscopic anterior cruciate ligament repair: A case report

**DOI:** 10.1016/j.tcr.2022.100672

**Published:** 2022-07-08

**Authors:** Elizabeth Simmons, Erin Sheffels, David Ulery, Amy Singleton, Nathan Fogt, Richard Miller

**Affiliations:** a Department of Orthopedic Surgery, Mercy Health-St. Vincent Medical Center, 2409 Cherry Street, MOB 1 Suite 10, Toledo, OH 43608, United States of America; b Superior Medical Experts, 1425 Minnehaha Ave E, St. Paul, MN, United States of America

**Keywords:** Pseudoaneurysm, All repair, Arthroscopic, Anterior tibial, Posterior horn

## Introduction

Arterial pseudoaneurysms are rare complications after arthroscopic anterior cruciate ligament (ACL) reconstruction, making up less than 0.3 % of all complications [1], [2]. ACL tears are among the most common orthopedic injuries, with an annual incidence of 68.6 per 100,000 persons as of 2010 [3]. Reconstruction rates have increased over time with up to 74.6 per 100,000 persons in 2014 [4]. The high incidence of ACL tears and reconstruction suggests that even rare complications, may affect a relatively large number of patients.

Pseudoaneurysms after ACL reconstruction have been reported as arising from the popliteal artery (Supplementary Table 1). While rare, a pseudoaneurysm can have severe consequences for the patient including neurological damage, deep venous thrombosis (DVT), and hemorrhage [5], [6], making management of risk factors and recognition of symptoms essential for increasing patient safety. Described below is a patient case demonstrating pseudoaneurysm formation from the anterior tibial artery after ACL reconstruction.

## Report of case

### Patient history and clinical findings

A 32-year-old woman injured her left knee after a trampoline accident, with immediate pain and inability to ambulate. In the emergency department (ED), swelling and tenderness of the knee along with radiographic imaging (X-ray) showing no fractures, dislocations, or acute osseous abnormalities (Supplementary Fig. 1) suggested a diagnosis of internal derangement of the left knee and ligamentous injury.

**Fig. 1:**
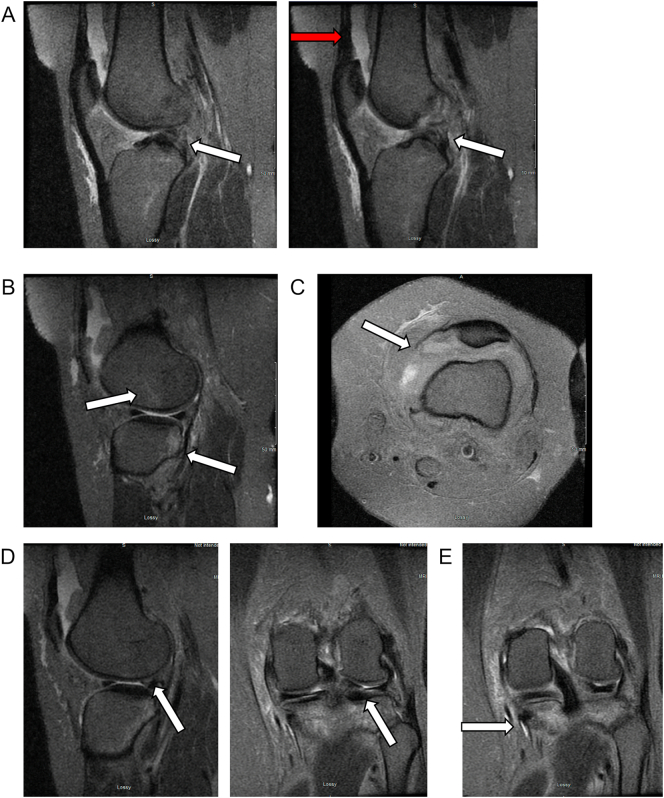
Sagittal cuts of magnetic resonance imaging (MRI) of the left knee demonstrating a vertical tear of the lateral meniscus, specifically the posterior horn and root of lateral meniscus extending through midportion. Moderate knee joint effusion is appreciated.

Two weeks post-injury, the patient's swelling in her left knee had subjectively improved but subjective buckling, pain with active knee motion, and difficulty with ambulation persisted. She had been using a patellar tracking orthosis (PTO) brace and crutches during ambulation. Magnetic resonance imaging (MRI) demonstrated evidence of complete anterior cruciate ligament (ACL) rupture, tears of the medial patellofemoral retinaculum, posterior horn and root of the lateral meniscus, and a grade 2 medial collateral ligament sprain (Fig. 1). After discussing treatment options, the patient elected to proceed with ACL reconstruction and meniscus repair.

### Initial surgery: arthroscopic anterior cruciate ligament reconstruction

Seven weeks after initial injury, the patient underwent arthroscopically assisted ACL reconstruction. Diagnostic arthroscopic demonstrated an incomplete medial meniscus tear, an empty lateral wall sign with the residual ACL attached to the tibia, and a lateral meniscal root tear with a complete posterior root avulsion tear.

A 270 mm semitendinosus allograft was quadrupled over with attachable buttons to both tibial and femoral sides for the ACL reconstruction. Tunnels for the graft were prepared using femoral and tibial guides in standard fashion with the tibial guide at 55°. After graft fixation, the knee was passively cycled through 25 repetitions of full range of motion (ROM) with stable repairs appreciated of the ACL. In addition, medial meniscus tear was fixed using an all-inside fixation technique by utilizing FiberLink (Arthrex, Naples, FL) sutures in a luggage-tag formation.

### Postoperative course: arthroscopic anterior cruciate ligament reconstruction

In the post-anesthesia care unit, the patient noted numbness and weakness in her bilateral lower extremities and she was diagnosed with right femoral nerve neuropraxia. Left leg numbness was attributed to the sciatic and femoral nerve blocks administered prior to ACL reconstruction procedure. The patient was discharged two days after surgery after her right lower extremity dysesthesias improved. At that time, she continued to have diffuse dysesthesias to the left leg with plan to observe outpatient.

Four weeks post-operatively, she was evaluated in the ED due to severe pain and swelling around the left knee. X-ray demonstrated no evidence of acute bony or joint space abnormalities (Supplementary Fig. 2) and Venous Doppler ultrasound failed to show evidence of DVT. She was subsequently discharged from the emergency department with instruction for outpatient follow-up. She presented for follow-up one week later, at which point a palpable fluctuance was noted over the anterolateral surgical incision with associated point tenderness, but no surrounding cellulitis or erythema was present. The fluctuance was aspirated, and 30 mL of hematogenous fluid was sent for pathology, with no concerning findings resulting.

One week after the aspiration the patient was again evaluated for weakness in the left foot. Along with new onset dysesthesias in the deep peroneal and superficial peroneal nerve distributions and decreased sensation on the plantar aspect of the left foot, there was a palpable firm painful mass on the anterolateral incision raising suspicion for an anterolateral hematoma compressing the peroneal nerve. An urgent MRI and electromyogram (EMG) were ordered but prior to completion of testing, the patient presented to the ED with new onset left foot drop, approximately six weeks after ACL reconstruction. At that time, there was a firm but compressible, non-tender swelling of the distal anterolateral incision, with a palpable pulsation (Fig. 3). Active ROM of the left knee was 0–70°. She was unable to perform straight leg raise, contract her tibialis anterior, extensor hallucis longus, or flexor hallucis longus. A bedside duplex ultrasound suggested hematoma associated with an unknown arterial branch. Computed tomography angiography (CTA) demonstrated a large pseudoaneurysm measuring 8.4 cm in diameter, originating from the left anterior tibial artery (Fig. 4).

### Pseudoaneurysm repair & postoperative course

**Fig. 2:**
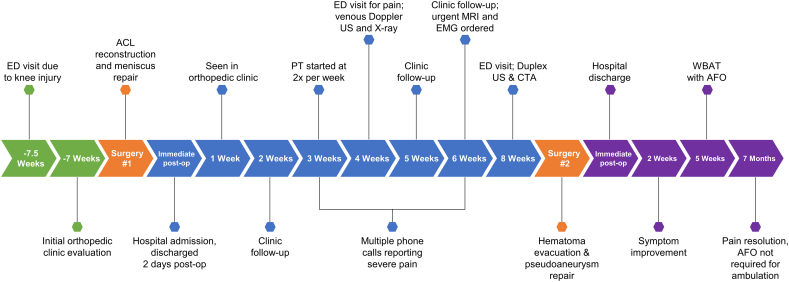
Timeline of treatment.

The vascular surgical team evacuated the hematoma and repaired the pseudoaneurysm without complication. Two weeks after pseudoaneurysm repair, she noted reduced pain and dysesthesias and restoration of toe motion of her left lower extremity. Twelve weeks after ACL reconstruction, the patient's foot motion continued to improve and she was allowed to bear weight as tolerated while wearing an ankle-foot-orthosis (AFO). Seven months after ACL reconstruction, the patient's knee pain had resolved and her foot drop had improved to where she no longer required an AFO although she did have a slight limp. A timeline of the patient's treatment course is outlined in Fig. 2.

**Fig. 3:**
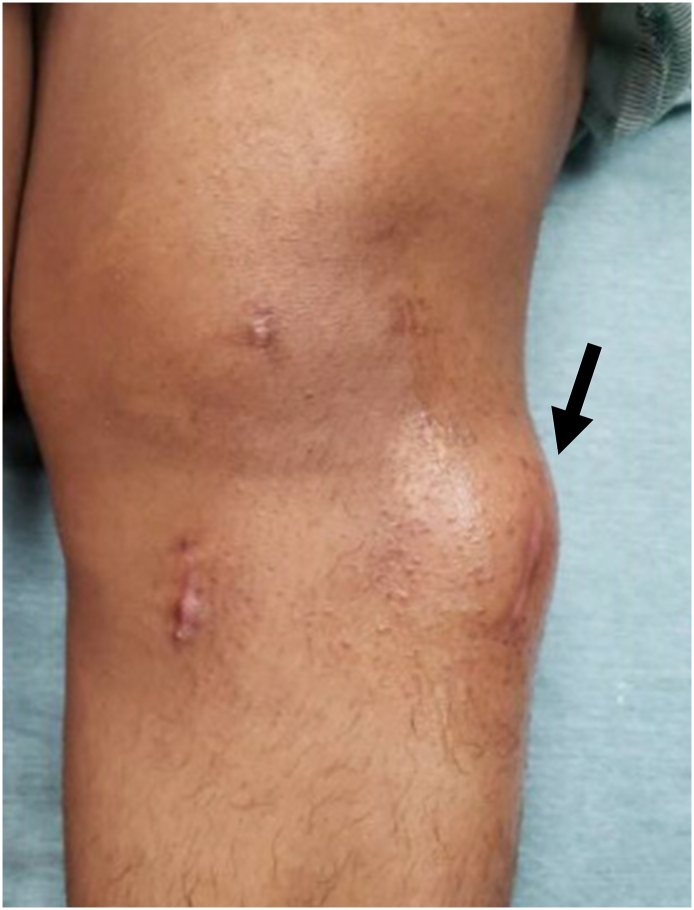
Clinical photograph from the day of pseudoaneurysm repair surgery. The large, fluctuant, pulsatile mass over the lateral surgical incision site is indicated with a black arrow.

**Fig. 4:**
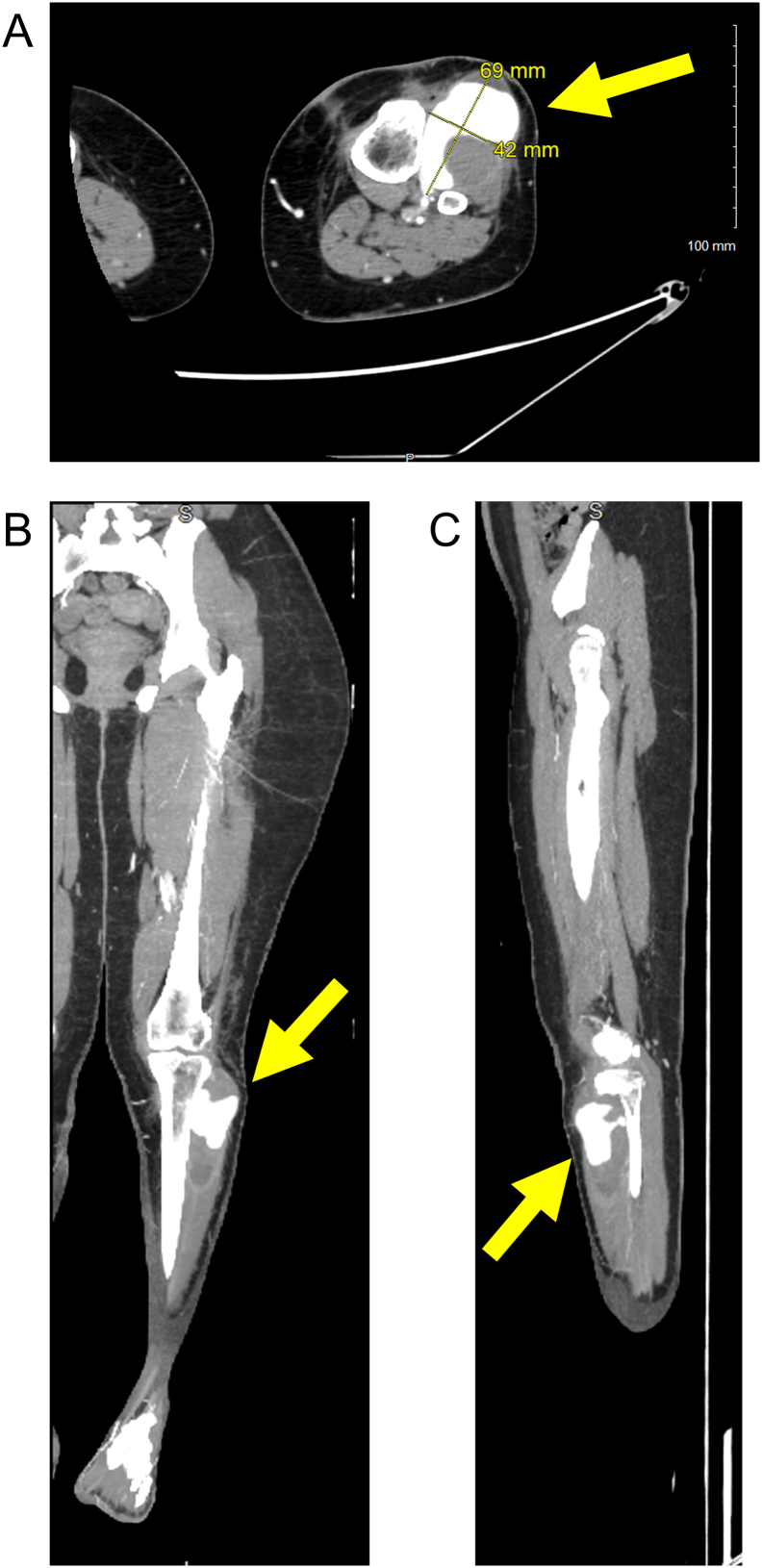
Computed tomography angiography of the left lower extremity demonstrating a large complex pseudoaneurysm arising from the anterior tibial artery, indicated with yellow arrows. A) Axial view, B) coronal view, and C) sagittal view.

## Discussion

Here we describe the first reported case of pseudoaneurysm arising from the anterior tibial artery after ACL reconstruction resulting in a foot drop, successfully treated with return to satisfactory ambulation at seven months.

In combined arthroscopic procedures, consideration of multiple etiologies of arterial damage must be considered, as injury could occur during both meniscectomy and general arthroscopic knee surgery [7]. Some pre-clinical studies have proposed techniques for preventing vascular damage during ACL repair [8], but further studies are needed to confirm that these strategies do not compromise the success of the repair. Aldrich et al. reported a case of pseudoaneurysm arising from the recurrent anterior tibial artery after meniscectomy [7], indicating that the arterial damage in our reported case could have been caused by the concurrent meniscus repair procedure as opposed to ACL reconstruction. The injury could have potentially also occurred during incision and/or dissection for placement of the guidewire and drill.

Pseudoaneurysms are rare but potentially devastating complications of ACL reconstruction [2], with clinical symptoms that include sensory deficits and painful pulsatile masses, which require prompt imaging. Similarly to previously reported cases, we noted a palpable dorsalis pedis pulse despite arterial injury and swelling, indicating the presence of distal pulses in the affected extremity is not an adequate diagnostic criterion to rule-out pseudoaneurysm. Prompt diagnosis and treatment limits the risk of permanent neurovascular consequences [1], [9], such as loss of sensation, deep vein thrombosis, and thromboembolism [5].

Insight into the presentation of pseudoaneurysms after ACL repair from this report and others may help decrease the time-to-diagnosis, decreasing the likelihood of lasting negative effects on the patient.

## Funding

No funding support was provided for this project.

## Ethics approval and consent to participate

The patient was informed that data from the case would be submitted for publication and gave her consent.

## Informed consent

The patient was informed that data from their case would be submitted for publication, and gave their informed consent.

## CRediT authorship contribution statement

Elizabeth Simmons contributed to the conception and design of the study, along with data collection. Erin Sheffels contributed to the study design and analysis and drafted the manuscript. David Ulery contributed to the study analysis and manuscript drafting. Amy Singleton contributed to study analysis, manuscript drafting/revisions, and administrative tasks. Nathan Fogt and Richard Miller contributed to study design and oversaw data collection. All authors reviewed and approved the final version of the manuscript.

## Declaration of competing interest

Erin Sheffels is employed by Superior Medical Experts. The authors declare no conflicts of interests concerning the materials or methods used in this manuscript.

## Appendix A Supplementary data

Supplementary material Image 1
