# Supplementary material for: Pseudoaneurysm in the anterior tibial artery after arthroscopic anterior cruciate ligament repair: A case report
Source: Trauma Case Rep. 2022 Jul 8;41:100672. doi: 10.1016/j.tcr.2022.100672 (PMC9287627; doi:10.1016/j.tcr.2022.100672)
Supplement: Supplementary file 1 — Supplementary material [file mmc1.docx]

**Supplementary Material**

**Pseudoaneurysm in the anterior tibial artery after arthroscopic anterior cruciate ligament repair: a case report**


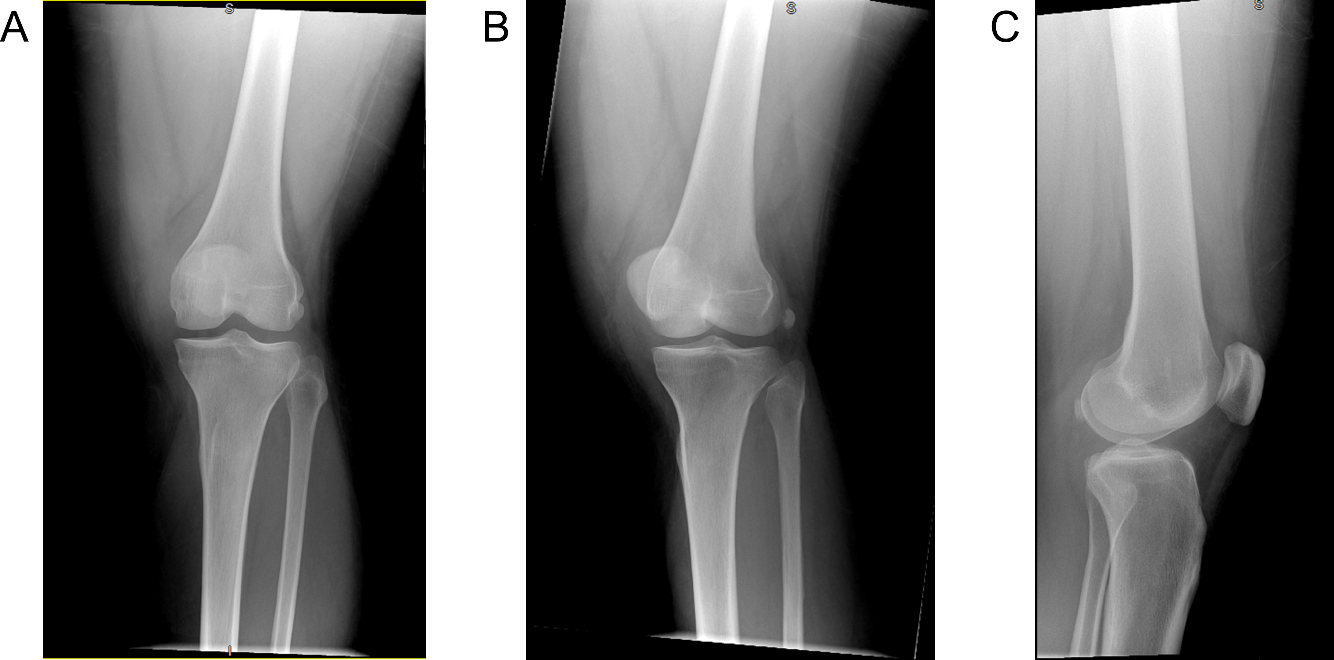


**Supplemental Figure 1.** X-ray of the left knee from the patient’s initial presentation to the emergency department demonstrating no acute fractures or dislocations, no focal osseous lesions, no joint effusions, and no obvious soft tissue abnormalities. A) anteroposterior view, B) oblique view, and C) lateral view


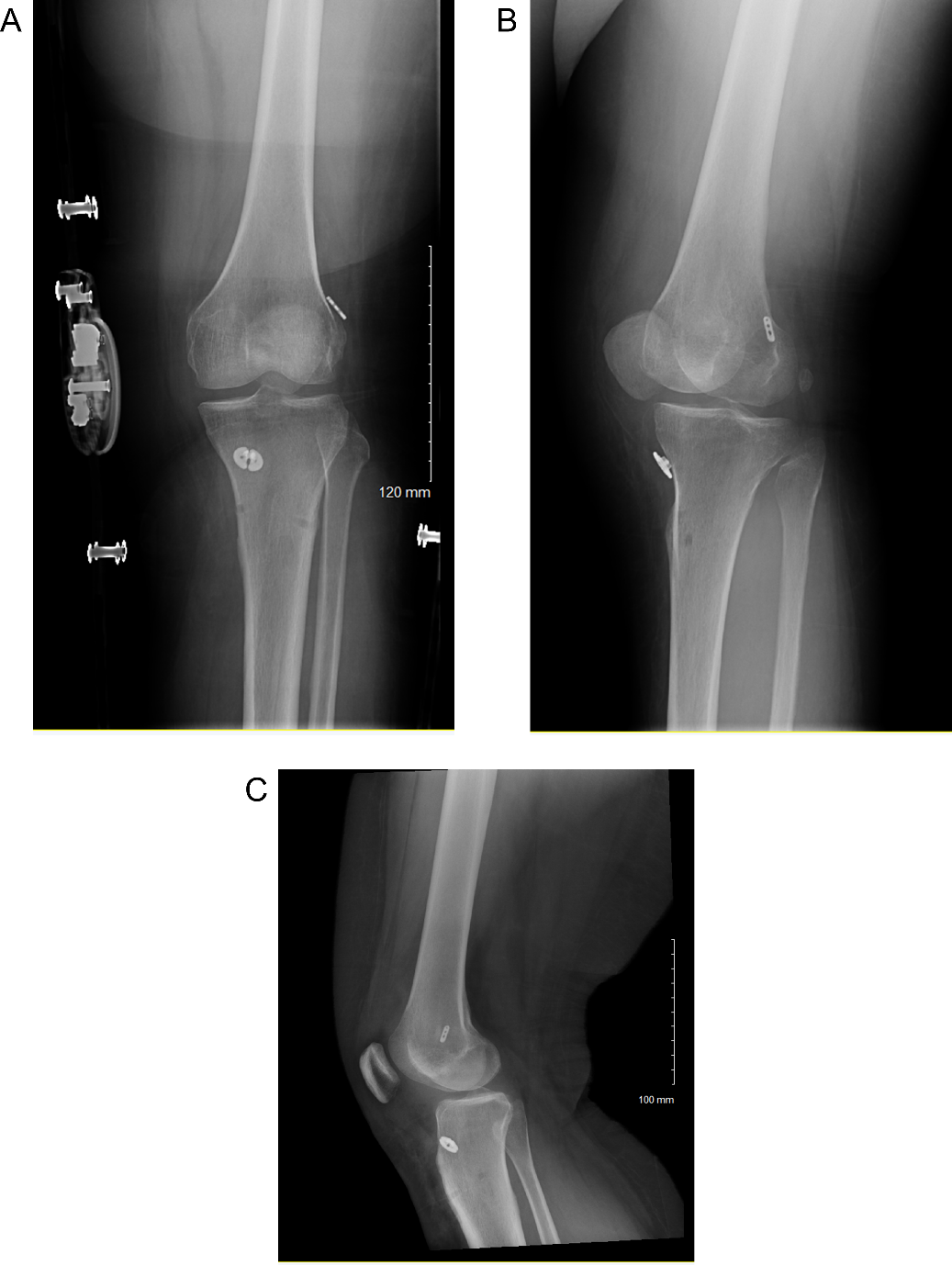


**Supplemental Figure 2.** X-ray images of the left knee from the patient’s emergency department visit four weeks after the initial surgery, demonstrating no acute fractures or dislocations, no focal osseous lesions, no evidence of joint effusion, and no soft tissue abnormalities. The ACL repair buttons are in place with no evidence of failure. A) anteroposterior view, B) oblique view, and C) lateral view

**Initial Surgery: Arthroscopic Anterior Cruciate Ligament Reconstruction**

The patient underwent an arthroscopically aided left ACL reconstruction, lateral meniscal root repair, and medial meniscus repair. Written consent was obtained prior to surgery. The patient received a pre-operative regional femoral nerve block and sciatic nerve block administered with ultrasound guidance in addition to general anesthesia. Antibiotics were infused prior to the start of surgery. An examination under anesthesia was performed prior to preparation and draping, which demonstrated an unstable anterior drawer, a grade 3A Lachman test, and a positive pivot shift. The posterior drawer and varus and valgus stress tests were negative.

After the exam, the tourniquet was inflated to 250 mmHg, standard sterile preparation and draping was completed, and the diagnostic arthroscopy was started. The arthroscopy began at the suprapatellar pouch, where there were no loose bodies. The patellofemoral joint had intact cartilage with no evidence of chondromalacia. No loose bodies were found in the lateral or medial gutters. After entering the medial joint and establishing a medial portal, a probe was inserted, which revealed an incomplete under-sided longitudinal tear in the central portion of the medial meniscus. The tear appeared on a white-red zone or red-red zone junction. After evaluating the full extent of the tear, the arthroscopy was continued by examining the notch. The examination revealed an empty lateral wall sign and the stump of the ACL attached to the tibia. The posterior cruciate ligament (PCL) was intact.

Moving to the lateral compartment revealed a lateral meniscal root tear with complete avulsion off the insertion of the lateral meniscus and the posterior root. Debridement of the lateral compartment was performed, including debridement of the insertion site with an arthroscopic shaver. A ball meniscal rasp was used to irritate the capsule and synovium to encourage bleeding for improved healing. The ACL insertion and stump origin and insertion stumps were also debrided. The meniscal repair was done by passing two sutures with a loop at one end (FiberLink) around the meniscal root in a luggage-tag formation. Then, the meniscal root repair guide was inserted and put in an appropriate position near the root insertion. A cannulated drill was used and when the appropriate positioning for pull-out sutures was confirmed, the cannulation was removed from the drill and a nitinol wire was advanced into the joint and pulled out through the cannula that was in place in the lateral port. The tails of the sutures in the meniscal root were placed through the loop in the nitinol wire and pulled through the tibial tunnel. The suture tails were retrieved through an incision on the lateral aspect of the anterior tibia. Final tensioning resulted in a well-reduced, anatomic root repair. The suture tails were secured to the tibia with a knotless anchor. The joint was examined with a scope and stable anatomic reduction was found.

Next, the medial meniscus was repaired using an all-inside fixation. Stable anatomic reduction and fixation of the medial meniscus was achieved. Concurrently, the allograft was prepared. A 280 mm semitendinosus allograft was used. The graft was trimmed to 270 mm and then quadrupled over with attachable button system buttons on the tibial and femoral sides. After the medial meniscus repair was finished, tunnels were prepared for ACL reconstruction. A femoral guide was used to ensure appropriate positioning of the femoral tunnel. To prepare the tibial tunnel, a tibial guide was used at 55 degrees. Again, a small hole was drilled to confirm positioning before expanding to a size appropriate for the graft.

After 140 minutes, the tourniquet was let down due to the maximum tourniquet time being reached. The tourniquet was re-inflated after 20 minutes, during which the graft preparation was finished. Next, graft insertion was started. The femoral side was inserted first and the seating of the button on the lateral cortex was performed prior to tensioning the femoral side. The tibial side was inserted, and the final tensioning of the femoral side was performed. The tibial side was also tensioned after confirming that the button was seated. After tensioning, the graft was put through 25 repetitions of full range of motion and tibial fixation was performed in full extension. Inspection of the joint showed an anatomic stable ACL reconstruction with appropriate tension. The meniscal repairs remained intact and appeared anatomic as well. The tourniquet was let down again, with a total tourniquet time of 176 minutes. No abnormal bleeding was noted. The incisions were closed, and the patient was put in a hinged knee brace locked in extension, with approval for a range of motion of zero to 90 degrees.

**Supplementary Table 1.** Pseudoaneurysm symptoms, diagnostic imaging, location, treatment, and outcome from previously reported cases of pseudoaneurysm after ACL repair

| **Study** | **Age/Sex** | **Symptoms of Pseudoaneurysm** | **Diagnostic Imaging** | **Surgical Intervention** | **Outcome** | **Full recovery?** |
| --- | --- | --- | --- | --- | --- | --- |
| **Pseudoaneurysm Location: Popliteal Artery** | | | | | | |
| Joshi et al. (2019) [1] | 32/M | Persistent posterior knee pain immediately post-op; foot drop and intense posterior knee pain developed at 4 week post-op | CTA, Doppler ultrasound | Exploration, excision of the pseudoaneurysm, and artery repair | Foot drop recovered at 3 months post-vascular surgery, return to sport at 1 year post-op | Yes |
| Ambrosia et al. (2015) [2] | 18/F | Popliteal artery spasm immediately post-op; foot paresthesias and weak pedal pulses at 7 weeks post-op | CTA, duplex ultrasound | Sustained balloon angioplasty for artery spasm; popliteal artery bypass with reverse saphenous vein autograft | Full recovery of knee function and neurovascular system by 8 months post-vascular surgery | Yes |
| Lee et al. (2014) [3] | 29/M | Posterior knee pain immediately post-op; increasing pain, swelling around popliteal area, and common peroneal nerve palsy starting one day post-op; continuing pain and swelling, muscle weakness, and sensory loss at 6 weeks post-op | CTA, MRI | Exploration, popliteal artery re-anastomosis with saphenous vein autograft | Posterior knee pain and swelling completely resolved and knee range of motion restored 3 months post-vascular surgery | Yes |
| Kanko et al. (2008) [4] | 23/M | Swelling in leg 1-month post-op; swelling in leg, discomfort and pain while walking, mild sensory loss on the foot, and weak distal pulses 2 years post-op | Doppler ultrasound, DSA | Exploration, pseudoaneurysm repair | Swelling, discomfort, and pain resolved; sensation restored to normal; no signs of arterial insufficiency | Yes |
| Janssen et al. (2004) [5] | 24/M | Sensory loss in lower leg at 3 days post-op; progressive posterior knee pain and additional sensory loss on foot at 12 days post-op | CTA, duplex ultrasound | Exploration, arterotomy, intimal lesion repair, venous patch closure of the arterial defect | Full motor function restored, sensory loss of saphenous and medial plantar nerves, mild sensory loss of superficial peroneal nerve at 4 months post-vascular surgery | No, mild sensory loss remaining at 4 months |
| Tam et al. (2014) [6] | 28/M | Acute swelling and dull ache in calf with diffuse ecchymosis around knee at 8 days post-op; acute compartment syndrome suspected leading to acute fasciotomy of the left calf and hematoma evacuation at unspecified time post-op | CTA, MRI | Arterial repair with saphenous vein graft | No sign of arterial insufficiency or edema immediately post-repair; patient able to follow usual ACL rehabilitation training | Yes |
| Galanakis et al. (2003)* [7] | 29/M | -- | Doppler ultrasound | -- | -- | -- |
| Xu et al. (2017) [8] | 57/M | Pain, swelling immediately post-op | CTA, Doppler ultrasound | Vascular stent implantation | Pain and swelling resolved by 7 days post-op | Yes |
| **Pseudoaneurysm Location: Posterior tibial artery** | | | | | | |
| Buda et al. (2008)* [9] | -- | Anemia at 5 days post-op | CTA | Vessel embolization | Anemia resolved, full recovery by 3 months post-embolization | Yes |
| **Pseudoaneurysm Location: Descending genicular artery** | | | | | | |
| Ho et al. (2020) [10] | 19/M | Large ecchymosis with focal swelling and tenderness around knee 6 days post-op; | CTA, Doppler ultrasound | Coil embolization, open debridement with drainage centered over the ecchymosis | No sign of arterial insufficiency or edema immediately post-op; full functional recovery and return to sport with mild paresthesia over the calf at 1 year post-embolization | Yes |
| Tsubosaka et al. (2017) [11] | 18/M | Bleeding 30 minutes post-op; pulsatile swelling of knee with no pain and normal distal pulses at 2 days post-op | CTA, Doppler ultrasound | Ultrasound-guided compression, microcatheter embolization | Full recovery with no complications by 1 year post-op | Yes |
| **Pseudoaneurysm Location: Medial inferior genicular artery** | | | | | | |
| Mello et al. (2011) [12] | 23/M | Acute hemarthrosis, drained and resolved 2 weeks post-op; repeated acute hemarthrosis 6 weeks post-op | MRA, MRI | Cyanoacrylate glue embolization | Full recovery with no neurovascular deficit by 5 years post-op | Yes |
| Milankov et al. (2006) [13] | 37/M | Continuous filling of the medial drain, swelling and pain on calf with normal distal pulses 1-hour post-op | CTA, Doppler ultrasound | Hematoma evacuation and blood vessel ligation | Full functional recovery and return to sport by 6 months post-op | Yes |
| Evans et al. (2000) [14] | 30/M | Swelling on knee at 4 weeks post-op; Pulsatile swelling with normal distal pulses at 5 weeks post-op | CTA, Doppler ultrasound | Exploration, blood vessel ligation, and thrombus removal from aneurysm sac | No evidence of recurrent aneurysm and return to physiotherapy at 2 weeks post-op; eventual full recovery | Yes |
| Filho et al. (2015) [15] | 17/M | Joint effusion and painful pulsatile mass in knee at 1 day post-op | Doppler ultrasound | Embolization | Full recovery with no recurrences | Yes |
| **Pseudoaneurysm Location: Lateral superior genicular artery** | | | | | | |
| Glanz (2020) [16] | 17/M | Intact neurovascular status at 3 days post-op; pulsatile painless mass on knee at 19 days post-op | CTA, Doppler ultrasound | Ultrasound-guided thrombin embolization | Full functional recovery and resolution of the pseudoaneurysm at 1 year post-op | Yes |
| Pereira et al. (2010) [17] | 20/M | Acute pain on knee, skin paleness, and edema with hematoma on thigh at 11 days post-op | Doppler ultrasound | Drainage of the hematoma and artery ligation | Eventual full functional recovery and return to physical activity | Yes |
|  | 38/M | Progressive pain and swelling on knee with normal distal pulses at 2 days post-op | Doppler ultrasound | Artery ligation | Absence of pain and return to physiotherapy protocol at 2 months post-op | Yes |
| Oversier et al. (2018) [18] | 25/M | Painful knee swelling at 3 weeks post-op | CTA, duplex ultrasound | Ultrasound-guided percutaneous thrombin injection, compressive bandage | No evidence of recurrent aneurysm at 20 weeks post-op | Yes |
| **Pseudoaneurysm Location: Deep femoral artery** | | | | | | |
| Fujii et al. (2016) [19] | 19/M | Knee pain and swelling on thigh at 7 days post-op | CTA, Doppler ultrasound, MRI | Ligation and resection of the pseudoaneurysm | Swelling and pain resolved immediately post-vascular surgery; full range of motion recovered at 3 months post-op; return to sports at 9 months post-op | Yes |

CTA=computed tomography angiography, DSA=digital subtraction angiography, MRA = magnetic resonance angiography, MRI=magnetic resonance imaging.

*Full text not available

**References:**

1. Joshi A, Singh N, Pradhan I, Basukala B. Pseudoaneurysm of the Popliteal Artery Leading to Foot Drop After Arthroscopic Anterior Cruciate Ligament Reconstruction: A Rare Complication: A Case Report. JBJS case connector. 2019;9(4):e0481. Epub 2019/10/15. doi: 10.2106/jbjs.Cc.18.00481. PubMed PMID: 31609751.

2. Ambrosia J, Qazi Z, Shuler FD, Giangarra C. Delayed Pseudoaneurysm of the Popliteal Artery Following ACL Reconstruction. Orthopedics. 2015;38(6):e543-6. Epub 2015/06/20. doi: 10.3928/01477447-20150603-93. PubMed PMID: 26091231.

3. Lee GC, Kim DH, Park SH. Popliteal artery pseudoaneurysm after anterior cruciate ligament re-revision using a rigidfix cross pin. Knee surgery & related research. 2014;26(2):121-4. Epub 2014/06/20. doi: 10.5792/ksrr.2014.26.2.121. PubMed PMID: 24944979; PubMed Central PMCID: PMC4061407.

4. Kanko M, Buluc L, Yavuz S, Muezzinoglu S, Berki T. Very rare aetiology of giant popliteal pseudoaneurysm: anterior cruciate ligament surgery. Postgraduate medical journal. 2008;84(989):158-9. Epub 2008/03/29. doi: 10.1136/pgmj.2007.062646. PubMed PMID: 18372488.

5. Janssen RP, Scheltinga MR, Sala HA. Pseudoaneurysm of the popliteal artery after anterior cruciate ligament reconstruction with bicortical tibial screw fixation. Arthroscopy : the journal of arthroscopic & related surgery : official publication of the Arthroscopy Association of North America and the International Arthroscopy Association. 2004;20(1):E4-6. Epub 2004/01/13. doi: 10.1016/j.arthro.2003.11.025. PubMed PMID: 14716289.

6. Tam K-WK, Wun Y-C, Lau C-Y, Chow Y-Y. Pseudoaneurysm of the Popliteal Artery after Anterior Cruciate Ligament Reconstruction Surgery: A Case Report and Literature Review. Journal of Orthopaedics, Trauma and Rehabilitation. 2014;18(1):44-6. doi: 10.1016/j.jotr.2013.12.001.

7. Galanakis I, Aligizakis A, Giannoukas A, Katsamouris A, Katonis P, Hadjipavlou A. Popliteal artery injury, peroneal nerve neurapraxia and tibial nerve transection of the right knee, after an arthroscopic assisted anterior cruciate ligament reconstruction. Minerva Ortopedica e Traumatologica. 2003;54:39-43.

8. Xu D, Ji L, Zhu J, Xie Y. Popliteal pseudoaneurysm and arteriovenous fistula after arthroscopic anterior and posterior cruciate ligament reconstruction: A case report. International journal of surgery case reports. 2017;40:50-3. Epub 2017/09/25. doi: 10.1016/j.ijscr.2017.07.056. PubMed PMID: 28941968; PubMed Central PMCID: PMC5609865.

9. Buda R, Massimiliano M, Caprio F, Ruffilli A, Rossi G, Giannini S. Pseudoaneurysm in a segmental branch of the posterior tibial artery following knee ligament reconstruction in a patient with favism. Medicina dello Sport. 2008;61:65-70.

10. Ho CW, Lee SH, Wu SH, Lin CY, Lee CH, Wu JL. Pseudoaneurysm following hamstring tendon harvest in arthroscopic anterior cruciate ligament reconstruction: a case report. BMC musculoskeletal disorders. 2020;21(1):697. Epub 2020/10/23. doi: 10.1186/s12891-020-03721-4. PubMed PMID: 33087086; PubMed Central PMCID: PMC7579808.

11. Tsubosaka M, Matsushita T, Kuroda R, Matsumoto T, Kurosaka M. Pseudoaneurysm of the articular branch of the descending genicular artery following double-bundle anterior cruciate ligament reconstruction. Knee surgery, sports traumatology, arthroscopy : official journal of the ESSKA. 2017;25(9):2721-4. Epub 2015/05/20. doi: 10.1007/s00167-015-3639-z. PubMed PMID: 25986096.

12. Mello W, de Brito WE, Migon EZ, Borges A. Pseudoaneurysm of the medial inferior genicular artery after anterior cruciate ligament reconstruction. Arthroscopy : the journal of arthroscopic & related surgery : official publication of the Arthroscopy Association of North America and the International Arthroscopy Association. 2011;27(3):442-5. Epub 2011/03/01. doi: 10.1016/j.arthro.2010.10.015. PubMed PMID: 21353173.

13. Milankov M, Miljkovic N, Stankovic M. Pseudoaneurysm of the medial inferior genicular artery following anterior cruciate ligament reconstruction with hamstring tendon autograft. The Knee. 2006;13(2):170-1. Epub 2006/02/07. doi: 10.1016/j.knee.2005.12.002. PubMed PMID: 16458515.

14. Evans JD, de Boer MT, Mayor P, Rees D, Guy AJ. Pseudoaneurysm of the medial inferior genicular artery following anterior cruciate ligament reconstruction. Annals of the Royal College of Surgeons of England. 2000;82(3):182-4. Epub 2000/06/20. PubMed PMID: 10858680; PubMed Central PMCID: PMC2503419.

15. Filho ES, Isolani GR, Baracho FR, de Oliveira Franco AP, Ridder Bauer LA, Namba M. Pseudoaneurysm after arthroscopic procedure in the knee. Revista brasileira de ortopedia. 2015;50(2):131-5. Epub 2015/08/01. doi: 10.1016/j.rboe.2015.03.001. PubMed PMID: 26229905; PubMed Central PMCID: PMC4519561.

16. Glanz L. Pseudaneurysm of the superolateral genicular artery following an anterior cruciate ligament reconstruction. International journal of surgery case reports. 2020;72:628-31. Epub 2020/07/03. doi: 10.1016/j.ijscr.2020.02.050. PubMed PMID: 32611535; PubMed Central PMCID: PMC7365774.

17. Pereira Junior EdS, Mestriner LA, Pereira EdS, Domingues RP, Cardoso MPA. Pseudoaneurisma de artéria genicular após cirurgia artroscópica de joelho: Relato de dois casos. Journal. 2010;18(2):104-6. doi: 10.1590/S1413-78522010000200009. (in Spanish).

18. Oversier LMR, Corten B, Barten DG, Elshof JWM. Pseudoaneurysm of the superior lateral genicular artery following anterior cruciate ligament repair. Acta chirurgica Belgica. 2018;118(4):258-63. Epub 2017/09/28. doi: 10.1080/00015458.2017.1371491. PubMed PMID: 28946810.

19. Fujii M, Furumatsu T, Kadota Y, Shimamura Y, Tsuchimochi S, Ozaki T. Pseudoaneurysm of the Perforating Branch of the Deep Femoral Artery Following Anterior Cruciate Ligament Reconstruction. Acta medica Okayama. 2016;70(6):515-8. Epub 2016/12/23. doi: 10.18926/amo/54818. PubMed PMID: 28003680.
